# Epidemiological, Gross Morphological, And Histopathological Analysis Of Postmortem Cases Of Hanging - An Observational Study

**DOI:** 10.12688/f1000research.159606.2

**Published:** 2025-02-17

**Authors:** Alana Chacko, Chandni Gupta, Vikram Palimar, Deepak Nayak M

**Affiliations:** 1 Department of Forensic Medicine, Kasturba Medical College, Manipal, Manipal Academy of Higher Education, Manipal, Karnataka, 576104, India; 2 Department of Anatomy, Kasturba Medical College, Manipal, Manipal Academy of Higher Education, Manipal, Karnataka, 576104, India; 3 Department of Pathology, Kasturba Medical College, Manipal, Manipal Academy of Higher Education, Manipal, Karnataka, 576104, India

**Keywords:** Hanging, Histopathology, Ligature mark, Postmortem cases, Vital Reaction

## Abstract

**Background:**

Death due to hanging is commonly seen. Sometime the ligature mark will be very prominent and in some cases it might not. In those cases, the post-mortem examination with its gross morphological findings and histopathological analysis, becomes significantly important. Objective: The objective of this study was to identify the patterns of epidemiological, gross morphological, and histopathological features in hanging cases.

**Methods:**

The study was conducted on 46 cases of hanging and was carried out from February 2023 to June 2024. Their sociodemographic features were collected. Gross morphological analysis of the ligature mark and various measurements were taken. Later tissue from the ligature mark was sent for histopathological analysis. Statistical analysis was performed on the collected parameters.

**Results:**

Significant male preponderance was observed in hanging cases. The maximum number of cases of hanging were observed in the fourth decade of life. Daily wage workers were the most significantly affected population. Depression was cited as the cause of suicide in most cases. Carotid intimal tears and injuries to bony and cartilaginous structures were present in smaller numbers. Vital reaction was observed in all 46 cases. Classification of wound vitality showed a correlation to known time since injury.

**Conclusion:**

The results of the present study showed that socio-demographic factors play a significant role in the circumstances leading to suicidal deaths. Therefore, in reducing the burden of suicidal deaths, these factors must be addressed. To identify the cause of death as hanging analysis of various gross features and histopathology are very important and should be conducted carefully.

## Introduction

Hanging is defined as a “form of asphyxia which is caused by the suspension of the body by a ligature which encircles the neck, the constricting force being the weight of the body”. ^1^ Based on the degree of suspension, hanging is classified into: 1) Complete hanging – Here the body is fully suspended without any part thereof touching the ground. 2) Partial hanging – Here the body is partially suspended with feet or knees touching the ground or the body assuming a sitting or kneeling position. ^1^ Based on the position of the knot in the ligature material, hanging is classified as: 1) Typical hanging – wherein the knot is present at the back of the neck, in the midline, and the ligature mark is bilaterally upward and symmetrical. 2) Atypical hanging – wherein the knot of the ligature material is present at any position other than the center of the back of the neck or occiput. ^1^

As per the 2021 and 2022 data of the National Crime Records Bureau, in India, hanging is the most adopted method of suicide, followed by a wide margin apart, poisoning, and drowning. ^2^ This may be due to the ease of accessibility of this method, and the potential of a less agonizing death. Instances of mass suicides, such as the Burari case, have also been characterized by mass suicidal hangings. Apart from judicial and suicidal hanging, lynching (of which one of the methods is homicidal hanging) has also been reported in various parts of India, including a case in Uttar Pradesh, where two Dalit girls were subjected to gang-rape and subsequently hung. ^3^

Various studies have been done over the years, analyzing the socio-demographic factors, the nature and cause of the act, gross findings on autopsy, and histopathology findings. These studies show non-uniform patterns concerning the parameters studied. ^4^^,^^5^

While regional differences may explain the variations in socio-demographic parameters, the marked variations in gross and histopathological findings cannot be attributed to the same. The question then arises of the best approach to autopsy in cases of hanging deaths, which best preserves and helps elicit the maximum number of findings, as well as helps arrive at a conclusive pathognomic finding in hanging cases. Hence, a more scientific approach should be implemented to assess the various techniques and signs observed.

The post-mortem examination with its gross morphological findings and histopathological analysis, becomes significantly important in cases where the characteristic ligature mark is inconspicuous or absent. In such cases, other marks of ante-mortem hanging are investigated. This includes the careful “bloodless” layer-by-layer dissection of the neck, looking for any features of injury, as well as the microscopic histopathological analysis of the skin from the suspected area of compression, to look for the ‘vital reaction’, which is currently the accepted ‘gold standard’. However, since the peri-mortem period and early post-mortem period injuries pose a significant challenge in microscopically assessing wound vitality, newer methods such as enzyme histochemistry and immunohistochemistry, are upcoming and in use.

Although advanced immunohistochemistry techniques yield significant results, the cost of the same and its practical application greatly limits its use. It is then that the routine microscopy comes back into the spotlight. Hence, there exists a need to identify a good predictive histopathological marker of antemortem injury and wound vitality in hanging cases.

Considering these facts, a knowledge gap exists, in the patterns of epidemiological, gross morphological, and histopathological features in hanging cases, where prior similar studies have not been conducted. Therefore, the present study was conducted to study the epidemiological profiles of autopsy cases of hanging, including socio-demographic patterns and psycho-social elements contributing to the manner of death, to study the gross morphological appearance of neck structures during external and internal examination, injuries and signs of asphyxia, in autopsy cases of hanging, to study the histopathological changes in skin and underlying tissues from the area of ligature mark and its adjacent areas, and to study histopathological determination of time since injury (wound vitality) in autopsy cases of hanging.

## Methods

It was a cross-sectional observational study. Institutional Ethical committee clearance (IEC:485/2022) was taken before starting the study from the Kasturba Medical College and Kasturba Hospital institutional ethics committee on 8 ^th^ February 2023.

We used the STROBE reporting guidelines for our study; a completed checklist is available under Reporting Guidelines. ^6^

The current research was carried out from February 2023 to June 2024 in the department of Forensic Medicine in association with the Department of Pathology.

Study was carried out on 46 Autopsy cases.

Cases of hanging deaths brought to Mortuary and cases in which the time of death has been established and is known were included in the study. Decomposed bodies were excluded.

Detailed description of procedure/processes:

Ethics committee has given the exemption from taking consent from the relatives of the deceased since these cases are postmortem cases where the law gives the consent for autopsy and determination of cause of death. In the above cases the samples were taken from death due to hanging so, the tissue must be taken out to determine the cause of death. The consent given by the law for autopsy is valid. The following details were collected from the police inquest documents, police intimation documents, bystanders of the deceased, and police:

1) Age, sex, occupation, marital status.
2) Case background, history of intoxication, place, date, and time of occurrence of the death.
3) Post-mortem number, date, and time of receiving the body in the mortuary.

## Procedure for external examination

**Figure 1.**
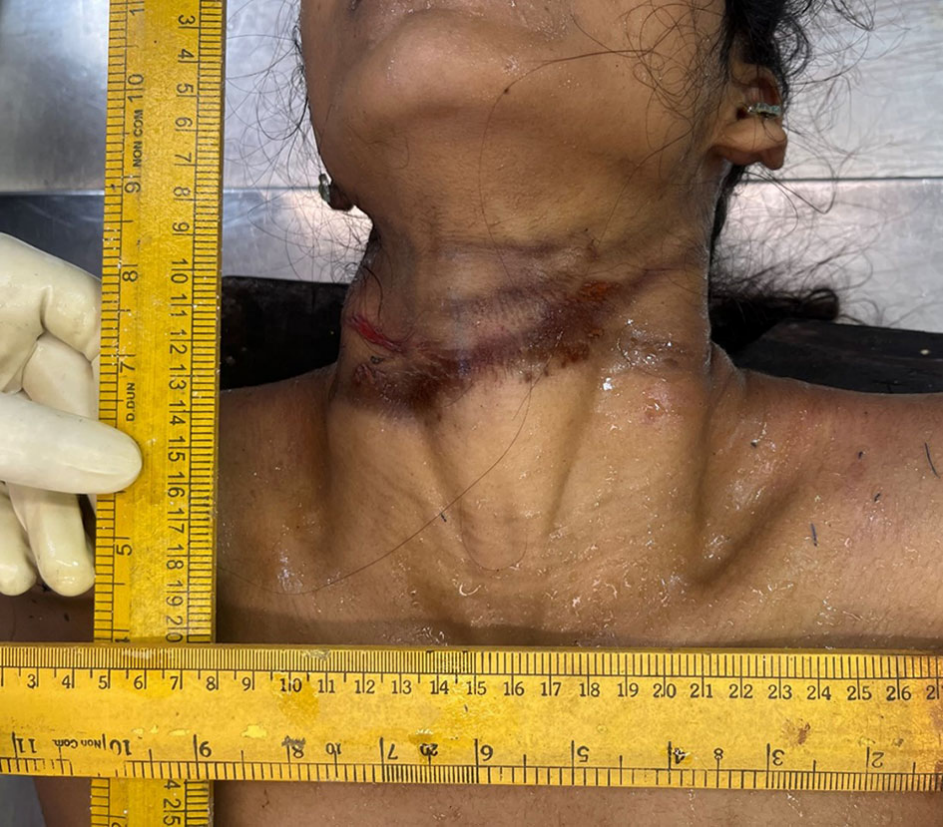
Showing ligature mark present at the level of thyroid cartilage.

Following the collection of the above data, and subject to fulfilment of inclusion criteria, the case was selected for the study and subjected to detailed post-mortem examination (autopsy). The external examination includes noting the presence or absence of ligature material in situ around neck. Type and manner of hanging was noted down. If present, type of ligature material used, type and position of knot, and any other significant features were noted, after which ligature material was secured with twine, ligated, and removed. Following this, clothes were examined and removed. The visible ligature mark was assessed for location with respect to the thyroid cartilage, and whether completely or incompletely encircling the neck was noted ( Figure 1). The rest of the general post-mortem external examination is conducted. After noting all the external features, the neck was dissected for internal examination.

## Procedure for neck dissection and internal examination

The linear incision for thorax and abdominal examination and evisceration is made from suprasternal notch to pubic symphysis, avoiding the umbilicus. A modification of Lettulle’s technique is used and evisceration is done, removing structures below the level of the lower end of the trachea corresponding to the sternoclavicular joint to the recto-sigmoid junction. Cranial dissection including stripping of dura is done, thus creating a bloodless field of dissection in the neck.

A 12-20 cm high block is placed underneath the shoulders, thus extending the neck for dissection. The skin incision is extended from the suprasternal notch to the symphysis menti. A 5 × 2 cm × striated muscle deep, tissue sample from the maximum compression area in the ligature mark, along with the tissue present above and below the ligature mark, is taken. The two latter areas are marked using India ink and the entire sample is preserved in 10% neutral buffered formalin and sent for histopathological examination. Using toothed forceps to hold the skin, dissection is done through the plane of the underlying subcutaneous tissue, up to the lower margins of the mandible, thus exposing the platysma muscle. The platysma muscle and deep cervical fascia are reflected to expose the muscles of the neck. The sternocleidomastoid muscle is identified, and its sternal and clavicular attachments are cut, and muscle is reflected upwards. This reveals the omohyoid muscle underneath. The inferior belly of the omohyoid muscle is cut and the muscle is reflected upwards, along with the median raphe and superior belly. The sternohyoid and the sternothyroid muscles are then identified and reflected upwards. This exposes the thyroid gland, which is dissected at the isthmus and reflected outwards to reveal the trachea underneath. Blunt dissection is used to separate the laryngeal apparatus, trachea, and pharynx from prevertebral tissue.

Opening the mouth, the tongue is pushed backward and upwards using forceps. Using a tongue knife inserted under the mandible, the attachment of muscles of the floor of the mouth and neck muscles are dissected. The dissection is continued backward, taking care to avoid the carotid arteries, and downwards up to the suprasternal notch and the pharynx and laryngeal structures are dissected out. The carotid arteries are divided in the neck and then removed. The hyoid bone is separated from its muscular attachments and examined. Later, the tissue was taken out for histopathological examination.

## Procedure for histopathology examination

The tissue was allowed to fixate for a minimum of eight hours in neutral buffered formalin. The tissue was dissected methodically beginning from the epidermis extending down to the dermis and the hypodermis. The sections were passed entirely and placed in tissue processor. The sections were embedded using paraplast as the embedding media. The section cutting was done using a 5-micrometre thickness and serial sections of the tissue (3 in number) were placed on the glass slide for staining. Staining was done by using hematoxylin and eosin (automated method). Special stains such as Masson trichrome (for collagen and fibrin) & elastic van Gieson (for elastin) were performed. The slides were mounted using DPX as the refractory medium. The slides were reviewed separately by a pathology resident and subsequently by a consultant, and observations were noted and classified according to Dettmeyer’s classification. ^7^

## Statistical analysis

Data was entered in Microsoft Excel sheet. Regular statistical analysis was done for the obtained parameters and results are presented in mean, frequency and percentage.

Underlying data is included in the Underlying Data section. ^8^

## Results

### Age

The age distribution of the deceased in the study ranges from 15 to 82 years. The mean age was 43.9 years. The maximum number of cases was observed in the age group of 41 to 50 years, with a significant male preponderance in this age group as Shown in Table 1.

**Table 1.** Sex-wise distribution of age groups.

Age group (in years)	Gender based frequency	Total	Percentage
10 – 20	Male: 1 Female: 2	3	6.5
21 – 30	Male: 7 Female: 2	9	19.5
31 – 40	Male: 5 Female: 2	7	15.2
41 – 50	Male: 11 Female: 1	12	26.1
51 – 60	Male: 3 Female: 1	4	10.9
61 – 70	Male: 6 Female: 1	7	10.9
71 and above	Male:4 Female: 0	4	10.9

### Gender

Among the deceased, the maximum number were males 37 (80.4%) when compared to females 9 (19.6%).

### Marital status

Of the deceased, 30 (65.2%) were married, 15 (32.6%) were unmarried and 1 (2.2%) were divorced. Among the married population, males were observed in more significant numbers (24 males).

### Occupation

The various occupations were classified into student, professional, clerical work, daily wage worker, housewife (homemakers), and unemployed. Among these, the highest incidence was among the daily wage workers (37%). The occupation-wise distribution is given in Table 2.

**Table 2.** Distribution of cases based on occupation.

Occupation	Frequency	Percentage
Student	6	13
Professional	1	2.2
Clerical work	8	17.4
Daily wage worker	17	37
Housewife (Homemaker)	6	13
Unemployed	8	17.4

### Place of occurrence

The case density based on location is given in Table 3.

**Table 3.** Distribution of cases based on location.

Location	Frequency	Percentage
Brahmavara	1	2.2
Gangolli	1	2.2
Hebri	3	6.5
Hiriyadka	8	17.4
Kapu	3	6.5
Karkala	1	2.2
Kota	1	2.2
Kundapura	1	2.2
Manipal	22	47.8
Padubidri	1	2.2
Udupi Town	4	8.7

### Associated intoxication

In 12 (26.1%) of the cases, there was associated intoxication by means of consumption of alcohol, of which the maximum number were found in the age group of 41 – 50 years. In 34 (73.9%) of cases there was no intoxication seen.

### Case background

Depression 19 (41.3%) was the most cited reason for suicide in both males and females, with the greatest number of cases recorded in the 21-30 years, 31-40 years and 41-50 years age groups (4 cases per group). This was followed by health issues 10 (21.7), family conflict 7 (15.2), financial issues 5 (10.9), psychiatric illness 4 (8.7%) and work stress 1 (2.2%).

### Signs of asphyxia

Congestion of the face was present in 15 (32.6%) of cases, and bluish discoloration of fingernails (cyanosis) was present in 42 (91.3%) of cases, however, oedema of the face was observed only in 2 (4.3%) of cases, and petechial haemorrhages were not observed in any cases.

### Signs of neck compression

Salivary dribbling was observed in 4 (8.7%) of cases whereas the characteristic ‘La facie sympathique was not observed in any cases.

### General post-mortem findings

Post-mortem lividity was observable in all cases, out of which, in 13 (28.3%) lividity was fixed and in 33 (71.7%), lividity was not fixed. Faecal stains alone were observed in 4 (8.7%) of cases, and seminal stains alone in 21 (45.7%) of cases. Both faecal and seminal stains were present in 5 (10.9%) of the cases. No urinary stains were observed in any of the cases. Subconjunctival haemorrhages were observed in 2 (4.3%) of the cases. 12 (26.1%) of the cases showed associated injuries.

### Features of ligature material and ligature mark

*Ligature material*

Out of the 46 autopsy cases, ligature material was absent during post-mortem examination in 5 of the cases. Therefore, they could not be assessed. The various ligature materials that were observed include nylon rope 19 (46.3%), saree 5 (12.2%), shawl 5 (12.2%), unrecognizable cloth 3 (7.3%), bedsheet 2 (4.9%), towel 2 (4.9%), dhothi 2 (4.9%), coir rope 1 (2.4%), electrical wire 1 (2.4%), and luggage belt 1 (2.4%).

### Type and position of knot

The ligature mark was found to run upwards and backwards from midline of the neck and was situated at a point above the thyroid cartilage in all the cases studied. Type and position of knot is shown in Tables 4 and 5.

**Table 4.** Distribution of type of knot.

Type of knot	Frequency	Percentage
Simple	2	5.4
Fixed	11	29.7
Running	24	64.9

**Table 5.** Distribution of position of knot.

Position of knot	Frequency	Percentage
Typical	5	14.3
Atypical	30	85.7

### Type and manner of hanging

All the autopsy cases of hanging in the study were suicidal in nature. 32 (69.6%) were cases of partial hanging while 14 (30.4%) of cases were complete hanging.

### Injuries to soft tissues and vasculature of the neck

The subcutaneous tissue in 44 (95.7%) of the cases was pale and glistening in nature, and unremarkable in 2 (4.3%) of the cases. Strap muscle contusions were observed in 2 (4.3%) of cases with one case showing contusion of both the sternohyoid and the sternothyroid muscle and one case of contusion of only the sternothyroid muscle.

Contusion of the sternocleidomastoid muscle was observed in 6 (13%) of the cases, all of them being cases of complete hanging. Out of these, most cases (3 in number) were those of atypical position of knot. Saree was used in two of the above cases. Other muscles of the neck were unremarkable in all cases studied.

Carotid intimal tears were noted in 3 (6.5%) of the cases, of which all were cases of complete hanging with atypical position of the knot of ligature material. Nylon rope, saree and unrecognizable cloth material were used in each of the cases. No instances of thyroid gland injuries were noted.

### Injuries to bony and cartilaginous structures of the neck

Hyoid bone and cervical vertebrae fractures were observed in 8 (17.4%) and 1 (2.2%) of the autopsy cases. Of the cases with hyoid bone fracture, most cases had an atypical position of the knot with nylon rope being used as the ligature material (4 cases). There was an even distribution of complete and partial hanging cases with hyoid bone fractures. No fractures were observed among the cartilages of the larynx.

Contusion of the hyoid bone was seen in 7 (15.2%) of cases, with most cases being those of complete hanging (5 cases), atypical position of knot (4 cases) and with nylon rope as the ligature material (4 cases). Contusion of thyroid cartilage was seen in 5 (10.9%) of cases, with distribution roughly like that of hyoid bone contusion. Contusions were not observed among other cartilages of larynx or cervical vertebrae.

### Vital reaction

In the tissue corresponding to the region of ligature mark, vital reaction was observed in 100% of cases. The regions above and below the region of ligature mark did not show features of extravasation of red blood cells or any features of vital reaction in any of the cases.

### Time since injury

**Figure 2.**
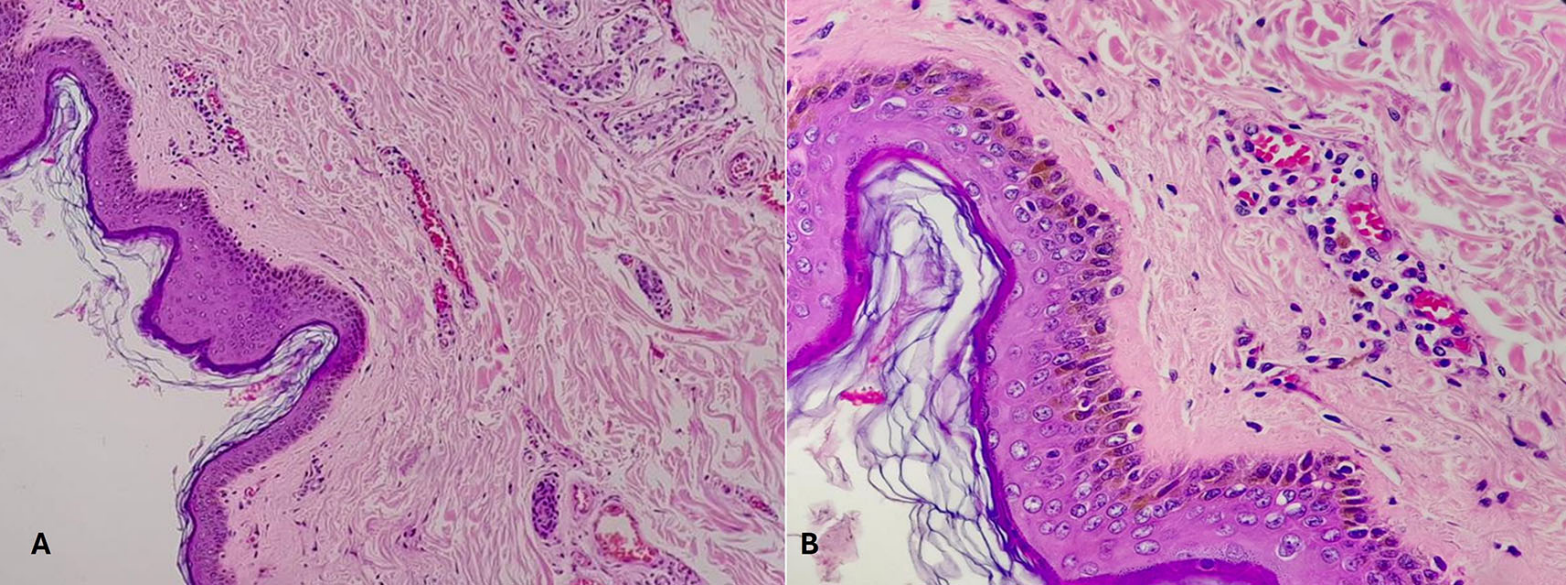
Wound incurred shortly, before or after death. (A) – H&E, 100×, (B) – H&E – 400×.

**Figure 3.**
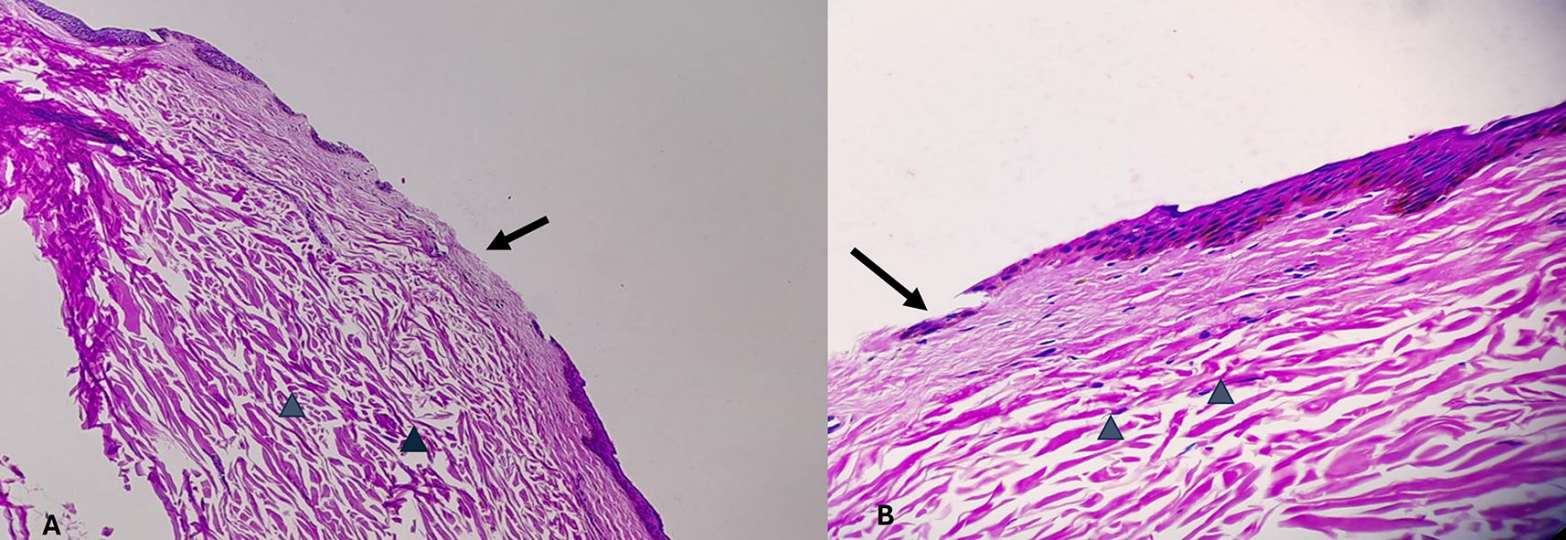
Vital wound-inflicted during lifetime. (A) – H&E, 100×, (B) – H&E – 400×.

**Figure 4.**
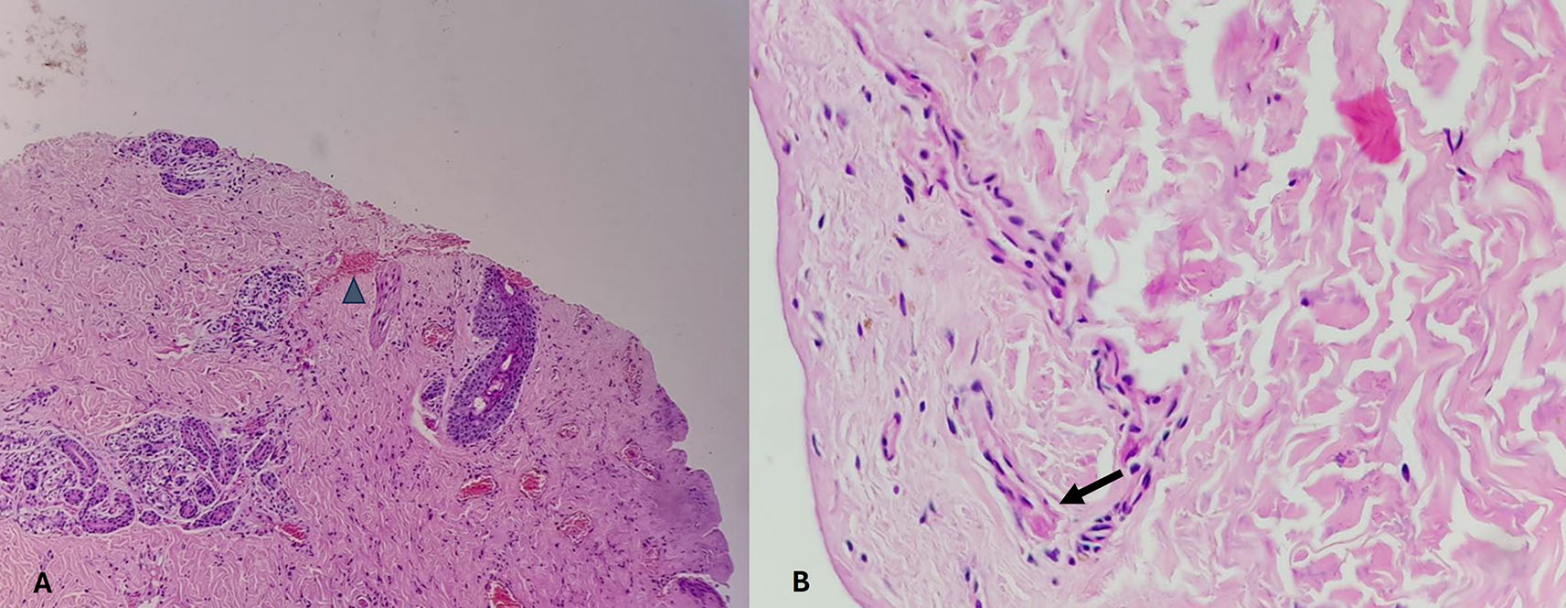
Fresh vital injury (hours to a few days). Fresh vital injury (hours to a few days). (A) – H&E, 100×, (B) – H&E – 400×.

**Figure 5.**
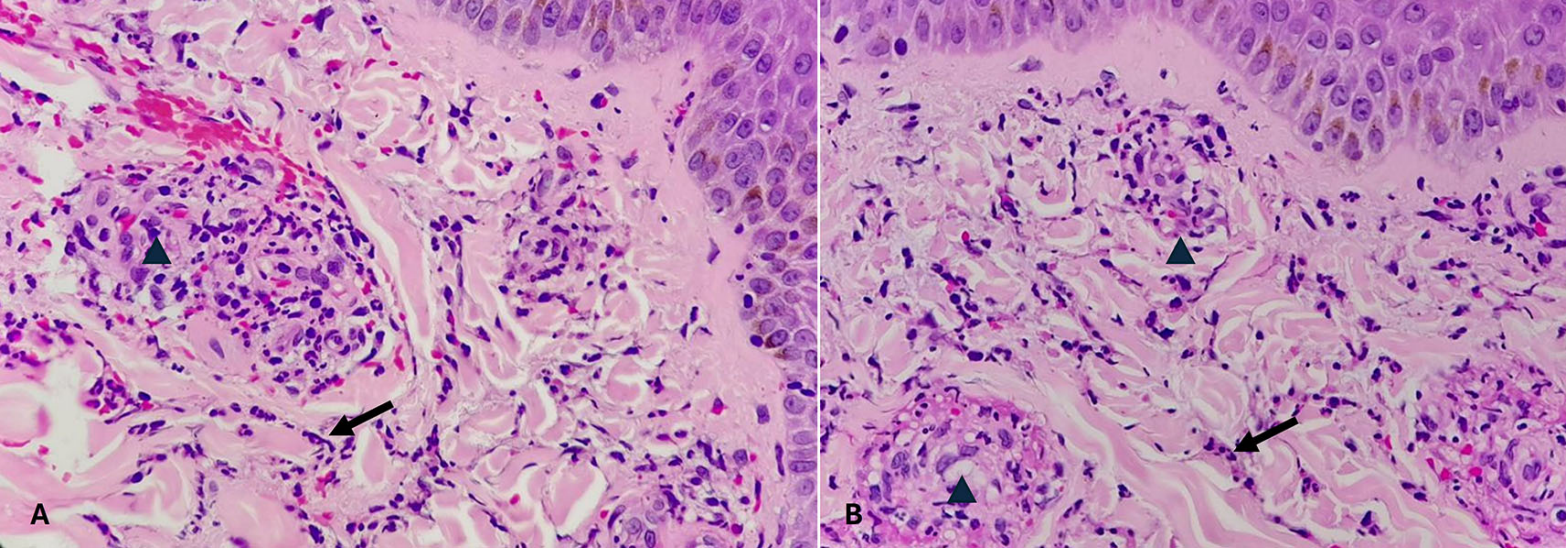
Vital wound-no longer fresh. (few days to weeks-in single digit range). (A) – H&E, 400×, (B) – H&E – 400×.

**Figure 6.**
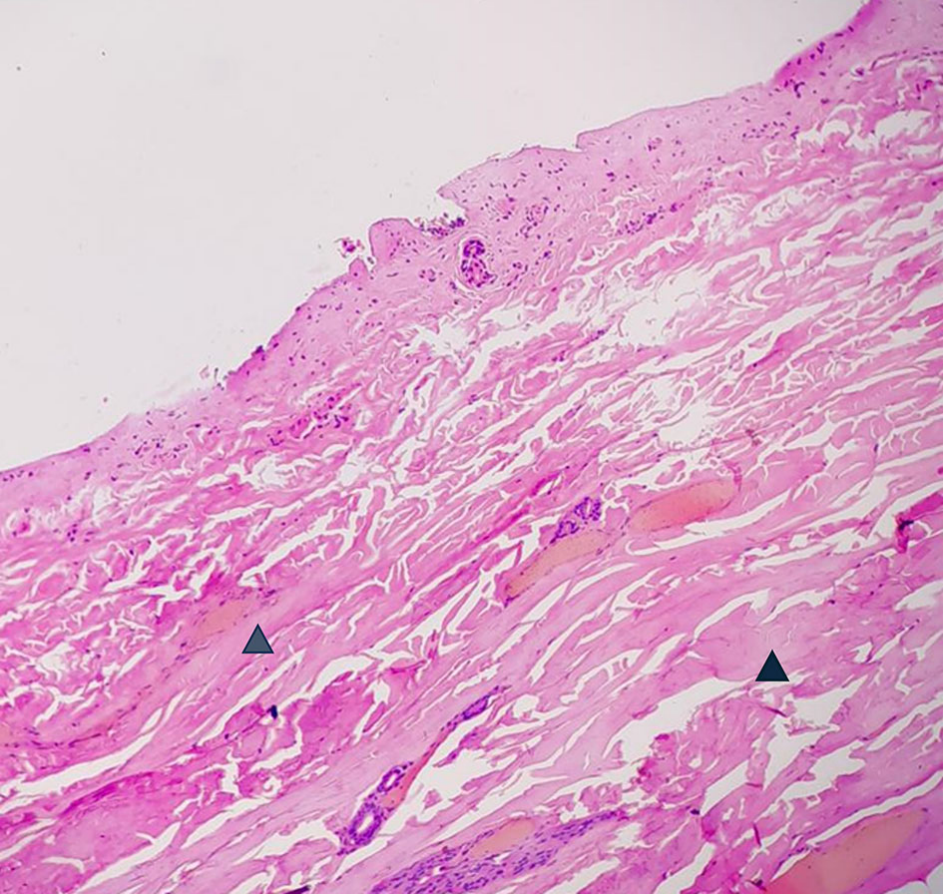
Vital Injury-Not very old (weeks to months). H&E, 100×.

Time since injury was broadly divided into fresh vital injury, vital wound, wound incurred shortly before or after death, not yet old and no longer fresh. The pattern of distribution of time since injury is given in the Table 6. Histopathological pictures of the tissues are shown in Figures 2- 6.

**Table 6.** Distribution of stages of time since injury.

Time since injury	Frequency	Percentage
Fresh vital injury	4	8.7
Vital wound	23	50
Wound incurred shortly before or after death	16	34.8
Not yet old	1	2.2
No longer fresh	2	4.3

Category 1. Wound incurred shortly, before or after death is shown in Figure 2. The epidermis is intact. The dermal collagen and vessels are unremarkable. No active immune reaction is noted. Category 2. Vital wound-inflicted during lifetime is shown in Figure 3. The epidermis is eroded (black arrow) and the dermal collagen shows disruption of fibroblasts (arrow heads). Category 3. Fresh vital injury (hours to a few days) is shown in Figure 4. The epidermis is eroded. The dermis shows a reaction with RBC extravasation (arrowhead) and vessels with fibrin thrombi (black arrow). Category 4. Vital wound-no longer fresh. (few days to weeks-in single digit range) is shown in Figure 5. The dermis shows a dense neutrophilic reaction (black arrow) with admixed mononuclear cells and macrophages, new vessel formation (arrow heads). Category 5. Vital Injury-Not very old (weeks to months) is shown in Figure 6. The epidermis is regenerating. Thick sclerosed collagen (arrow heads), representing scar tissue; with loss of dermal adnexal structures and scant inflammation.

## Discussion

Violent deaths resulting from asphyxia predominantly involve hanging. Medico-legal issues likely to develop in case of hanging are primarily, whether the death was caused by hanging was suicidal, homicidal or accidental. Simulated suicidal hanging hinders the investigation procedure in unnatural deaths. Therefore, detailed external and internal examination, and analysis of samples play a significant role in arriving at conclusion. Apart from postmortem the ligature material used, location, point of suspension and evaluation of scene of crime might add to the inference. ^9^

Previous studies associating gender connotations with hanging as a method of suicide have found that men were most found to adopt this method, as observed in studies by Denning et al. and Mergl et al. ^10, 11^ In studies done in the state of Karnataka, similar findings were observed concerning the male preponderance in hanging cases. ^4, 12^ The findings in the current study are comparable to the above-referenced studies, with a male-to-female ratio of 4.1:1. However, this ratio is more than two-fold higher as compared to the various other studies by Dekal et al., Haq et al., Karthik et al., Kumar et al, and Biradar et al. in Karnataka, wherein the ratio was only 1.5:1 to 1.13:1. It was also observed that the highest number of cases occurred in the age group of 41 to 50 years, which deviates from the findings in the above studies. ^5, 13– 16^

It was also observed that among the deceased, the population of married people was significantly higher than the unmarried population, which was comparable to similar studies done by Karthik et al. ^14^

Depression was the leading cause of suicide by hanging in the present study (41.3%), followed by health issues (21.7%), and family conflict (15.2%). This finding becomes significant due to the reason that the previously cited various other studies have not attributed the cause of suicides by hanging to depression. Yang et al. studied the trends of depressive disorder over a period of 30 years in 204 countries and found that depression was one of the leading causes global disease burdens. ^17^ Hence, this finding becomes more harrowing and points to the need for stepping up efforts in mental health programs.

Though it has been suggested that ‘asphyxial stigmata’ or signs of asphyxia have become redundant findings in forensic practice, they are still observed and recorded in routine autopsies. ^18^ This is because, although they may be inconclusive findings, they still direct the examiner towards a particular modality of death, especially in cases with no visible cause of death or reliable history. The study by James and Silcocks observed petechial hemorrhages in 23% of cases and congestion of face in 5% of cases. ^19^ Lockyer BE found an average incidence of 25% for petechiae. ^20^ In this study, congestion of the face was noted in 32.6% of cases and cyanosis in 91.3% of cases, comparable to existing literature. However, edema of the face and subconjunctival hemorrhages were observed only in 4.3% of cases, and petechial hemorrhages were not observed.

Classically described signs, such as salivary dribbling and ‘la facie sympathique, were low (4.3%) and absent respectively, in the study. This contrasts with existing literature. ^1, 21^ Deaths due to hanging are often associated with involuntary urination, defecation, or ejaculation. ^1^ The present study found 45.7% of cases to have seminal stains, 8.7% of cases to have fecal stains and 10.9% of cases having both seminal and fecal stains. However, urine stains were not observed in any of the cases.

Examination of the subcutaneous tissue revealed it to be pale and glistening in 95.7% of cases, which was comparable to previous studies by Suarez-Penaranda et al., Jiwane et al., and Simonsen. ^22– 24^ 4.3% of cases showed contusion to strap muscles of the neck and 13% of cases showed contusion to the sternocleidomastoid muscle, which are comparable to previous studies ^20, 22, 23, 25^

Carotid intimal tears (Amussat’s sign) were observed in 6.5% of cases, comparable to existing trends in previous studies. ^26^

The incidence of hyoid bone contusion (15.2%), hyoid bone fracture (17.4%), and thyroid cartilage contusion (10.9%), were significantly lower than in previous studies by Eisenmenger and Betz, Suarez-Penaranda, and Nikolic et al. ^22, 27, 28^ However, they were comparable to studies by Kokatanur et al. and Feigin G. ^29, 30^ The incidence of cervical vertebrae fracture (2.2%), was comparable to studies by Suarez-Penaranda et al. and Jayaprakash et al. ^22, 31^

In the current study, vital reactions (including features such as hemorrhage, inflammatory self- infiltration) were observed in all the cases included in the study. These findings were in alignment with those from studies by Kokatanur et al. and Prasad et al. ^30, 32^

### Limitations of the study

1) Limited number of cases for study in the study period.
2) Advanced enzyme histochemistry and immunohistochemistry techniques were not used, which can be done in future studies.

## Conclusion

This study was an attempt to bridge the existing knowledge gap in suicidal cases of hanging. It is evident from this study that socio-demographic factors play a significant role in the circumstances leading to suicidal deaths. Therefore, in reducing the burden of suicidal deaths, these factors must be addressed. The analysis of various gross features shows that the findings in existing literature are not always observed practically and in real-world situations. Finally, further research on the estimation of wound vitality and a consensus on the definition of the term ‘vital reaction’ will be of great forensic value.

### Ethical considerations

Ethical clearance was taken from the Kasturba Medical College and Kasturba Hospital institutional ethics committee on 8 ^th^ February, 2023. Approval no: (IEC:485/2022).

## Data Availability

Figshare: Epidemiological, Gross Morphological, And Histopathological Analysis Of Postmortem Cases Of Hanging - An Observational Study. https://figshare.com/articles/dataset/Data_sheet/27917280?file=51057245. ^6^ The project contains the following underlying data: Data sheet of all 46 cases. Data are available under the terms of the Creative Commons Zero “No rights reserved” data waiver (CC0 1.0 Public domain dedication). Figshare: Epidemiological, Gross Morphological, And Histopathological Analysis Of Postmortem Cases Of Hanging - An Observational Study. https://figshare.com/articles/online_resource/Strobe_checklist/27917358?file=50835189. ^8^ Data are available under the terms of the Creative Commons Zero “No rights reserved” data waiver (CC0 1.0 Public domain dedication).
