# Supplementary material for: Epidemiological, Gross Morphological, And Histopathological Analysis Of Postmortem Cases Of Hanging - An Observational Study
Source: F1000Res. 2025 Feb 17;13:1556. Originally published 2024 Dec 24. [Version 2] doi: 10.12688/f1000research.159606.2 (PMC11845901; doi:10.12688/f1000research.159606.2)
Supplement: Peer review history

# Reviewer report: Reviewer response for version 2

**DOI:** 10.5256/f1000research.177755.r402940

**Published:** 2025-08-20

**Authors:** Jayanthi Yadav

**Affiliations:** 1 All India Institute of Medical Sciences, Bhopal, India

1. The authors have indicated that ethical approval was obtained (IEC:485/2022) and that an exemption from family consent was granted, since only tissues routinely collected during medicolegal autopsy were used, with no additional sampling performed. This clarification appropriately addresses the ethical aspect. It may still be helpful for the authors to state this more explicitly in the manuscript, so that readers clearly understand the basis of the waiver.

 2. While the study is meticulously conducted, the manuscript would benefit from improved clarity in writing and organization.

 3. The authors mentioned about % of partial /complete hanging but nowhere is it mentioned how tis was established? Did they rely on autopsy findings / History from police /or they had crime scene photographs? 

 4. The authors mention about thyroid and hyoid fracture but neither in the results / Discussion the incidence of fracture has been correlated with age of the deceased. 

 5. What parameters were used to establish vitality of wounds in histopathology. details are missing.

 6. Age of injury has been described with figures in details but paradoxically, the type and location of injury id blatantly missing.

 7. How the injuries are related to the hanging is also not explained. I found the description of injuries totally out of place for this manuscript. 

 8. In discussion the authors have only compared the incidence of their findings with other authors but have not discussed anything concrete to support their findings.

Is the work clearly and accurately presented and does it cite the current literature?

Partly

If applicable, is the statistical analysis and its interpretation appropriate?

Not applicable

Are all the source data underlying the results available to ensure full reproducibility?

No

Is the study design appropriate and is the work technically sound?

Yes

Are the conclusions drawn adequately supported by the results?

Partly

Are sufficient details of methods and analysis provided to allow replication by others?

Partly

Reviewer Expertise:

Forensic Medicine & toxicology

I confirm that I have read this submission and believe that I have an appropriate level of expertise to confirm that it is of an acceptable scientific standard, however I have significant reservations, as outlined above.

# Reviewer report: Reviewer response for version 2

**DOI:** 10.5256/f1000research.177755.r367044

**Published:** 2025-02-21

**Authors:** K R Nagesh

**Affiliations:** 1 Father Muller Medical College, Mangalore, India

The authors incorporated the changes suggested. Hence, I approve this manuscript.

Is the work clearly and accurately presented and does it cite the current literature?

Partly

If applicable, is the statistical analysis and its interpretation appropriate?

Partly

Are all the source data underlying the results available to ensure full reproducibility?

Yes

Is the study design appropriate and is the work technically sound?

Yes

Are the conclusions drawn adequately supported by the results?

Partly

Are sufficient details of methods and analysis provided to allow replication by others?

Yes

Reviewer Expertise:

Forensic Pathology, Forensic Anthropology, Forensic Odontology, Forensic Toxicology

I confirm that I have read this submission and believe that I have an appropriate level of expertise to confirm that it is of an acceptable scientific standard.

# Reviewer report: Reviewer response for version 1

**DOI:** 10.5256/f1000research.175362.r354475

**Published:** 2025-01-31

**Authors:** K R Nagesh

**Affiliations:** 1 Father Muller Medical College, Mangalore, India

Authors have analyzed the demographic details in fatal hanging cases during the study period. The following are my suggestions.

**Abstract: **

- The duration of the study needs to be added in the methods.
- In results section, the last sentence needs spelling correction (wound vitality).

**Introduction:**

- The first three paragraphs (definition and classification of hanging) can be combined and put under one paragraph, and reference needs to be added wherever is appropriate.
- Various studies have been done over the years … Reference needs to be added.

**Method:**

- It is better to write the initial part of methods (study type to exclusion criteria) in descriptive manner.
- Type of study can be changed to ‘Cross-sectional observational study’.
- Statistical analysis: The statistical tool used to enter data and type of analysis done needs to be added.

**Conclusion:**

- Conclusion needs to be added after discussion.

**Strengths:**

 Authors have studied the socio-demographic findings related to hanging cases during the study period, and analyzed the gross and histopathological findings seen at ligature mark in hanging cases. The results are well presented.

**Weakness:**

 Sample size is less and authors have mentioned under limitations. The writing part under few sections as mentioned above needs to be addressed.

**Recommendation:**

 I recommend for ‘Acceptance after undertaking the above corrections’.

Is the work clearly and accurately presented and does it cite the current literature?

Partly

If applicable, is the statistical analysis and its interpretation appropriate?

Partly

Are all the source data underlying the results available to ensure full reproducibility?

Yes

Is the study design appropriate and is the work technically sound?

Yes

Are the conclusions drawn adequately supported by the results?

Partly

Are sufficient details of methods and analysis provided to allow replication by others?

Yes

Reviewer Expertise:

Forensic Pathology, Forensic Anthropology, Forensic Odontology, Forensic Toxicology

I confirm that I have read this submission and believe that I have an appropriate level of expertise to confirm that it is of an acceptable scientific standard, however I have significant reservations, as outlined above.

# Response

**Published:** 2025-01-31

**Authors:** Vikram Palimar

**Affiliations:** Forensic Medicine, Manipal Academy of Higher Education, Manipal, Karnataka, India

**Abstract:**

- The duration of the study needs to be added in the methods.
**Response **- Added
- In results section, the last sentence needs spelling correction (wound vitality).
**Response **- Corrected

**Introduction:**

- The first three paragraphs (definition and classification of hanging) can be combined and put under one paragraph, and reference needs to be added wherever is appropriate.
**Response **- Combined and same reference 1 was there which was added in between. 
- Various studies have been done over the years … Reference needs to be added.
**Response **- Added the references.

**Method:**

- It is better to write the initial part of methods (study type to exclusion criteria) in descriptive manner.
**Response **- Written in descriptive form.
- Type of study can be changed to ‘Cross-sectional observational study’.
**Response **- Changed the study type. 
- Statistical analysis: The statistical tool used to enter data and type of analysis done needs to be added.
**Response **- Added

**Conclusion:**

- Conclusion needs to be added after discussion.
**Response **- Added

# Reviewer report: Reviewer response for version 1

**DOI:** 10.5256/f1000research.175362.r354484

**Published:** 2025-01-17

**Authors:** Priyadarshee Pradhan, Sneha S

**Affiliations:** 1 Sri Ramachandra Institute of Higher Education and Research (Deemed to be University), Chennai, Tamil Nadu, India; 2 Forensic Medicine & Toxicology, Sri Ramachandra Institute of Higher Education and Research (Deemed to be University) (Ringgold ID: 204733), Chennai, Tamil Nadu, India

1. The type of observational study can be specified under study type in the Methods section ( Suggestion - cross sectional study)

 2. In the last paragraph of introduction, it is mentioned that no similar studies have been conducted in the past.

 How did the authors ensure this statement?

 Suggestion: Can give an explanation or avoid the phrase.

 3. How did the authors arrive at a sample size of 46?

Is the work clearly and accurately presented and does it cite the current literature?

Yes

If applicable, is the statistical analysis and its interpretation appropriate?

Yes

Are all the source data underlying the results available to ensure full reproducibility?

Yes

Is the study design appropriate and is the work technically sound?

Partly

Are the conclusions drawn adequately supported by the results?

Yes

Are sufficient details of methods and analysis provided to allow replication by others?

Yes

Reviewer Expertise:

Forensic Pathology, Forensic Anthropology

We confirm that we have read this submission and believe that we have an appropriate level of expertise to confirm that it is of an acceptable scientific standard.
